# Incidentally discovered tracheal bronchus

**DOI:** 10.1002/rcr2.1247

**Published:** 2023-11-22

**Authors:** Chia‐Wei Chang, Chun‐Han Wu

**Affiliations:** ^1^ Division of Pulmonary Medicine, Department of Internal Medicine Tri‐Service General Hospital, National Defense Medical Center Taipei Taiwan, ROC

**Keywords:** airway, anomalies, bronchus, congenital, tracheal

## Abstract

Congenital anomalies of the large airways are occasionally asymptomatic and are incidentally discovered through radiography, often using computed tomography. Bronchoscopy can aid in the direct visual examination of the large airway abnormalities detected on radiography.

## Graphical abstract

Congenital anomalies of the large airways are occasionally asymptomatic and are incidentally detected using radiography. Although most patients are asymptomatic, clinical conditions such as bronchial stenosis, stridor, cough, recurrent episodes of pneumonia, or malposition of the endotracheal tube may occur. Physicians should be aware of this anomaly and its possible complications.

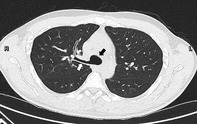

## CLINICAL IMAGE

**FIGURE 1:**
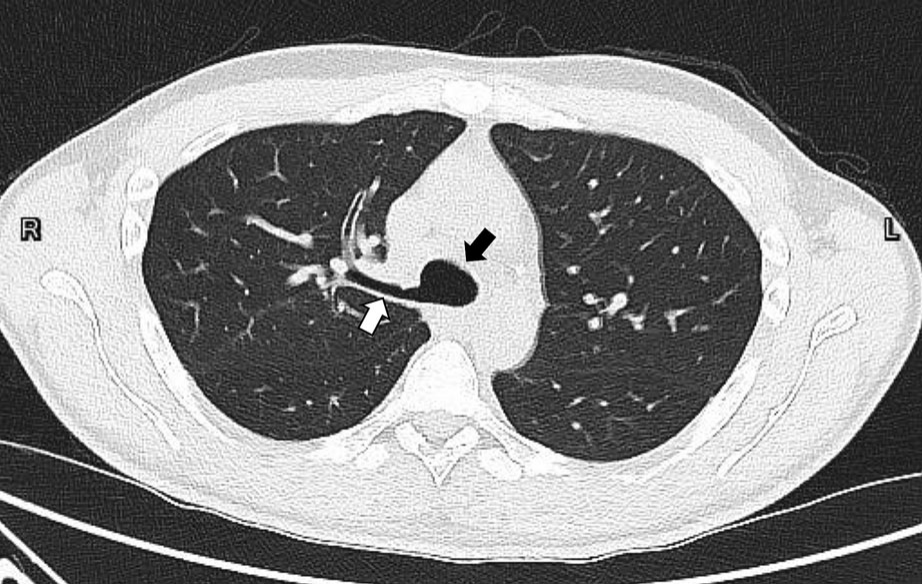
Axial view of chest computed tomography with lung window revealing tracheal bronchus (white arrow) over the right‐sided distal tracheal (black arrow) above the carina with absence of right upper lobe bronchus.

**FIGURE 2:**
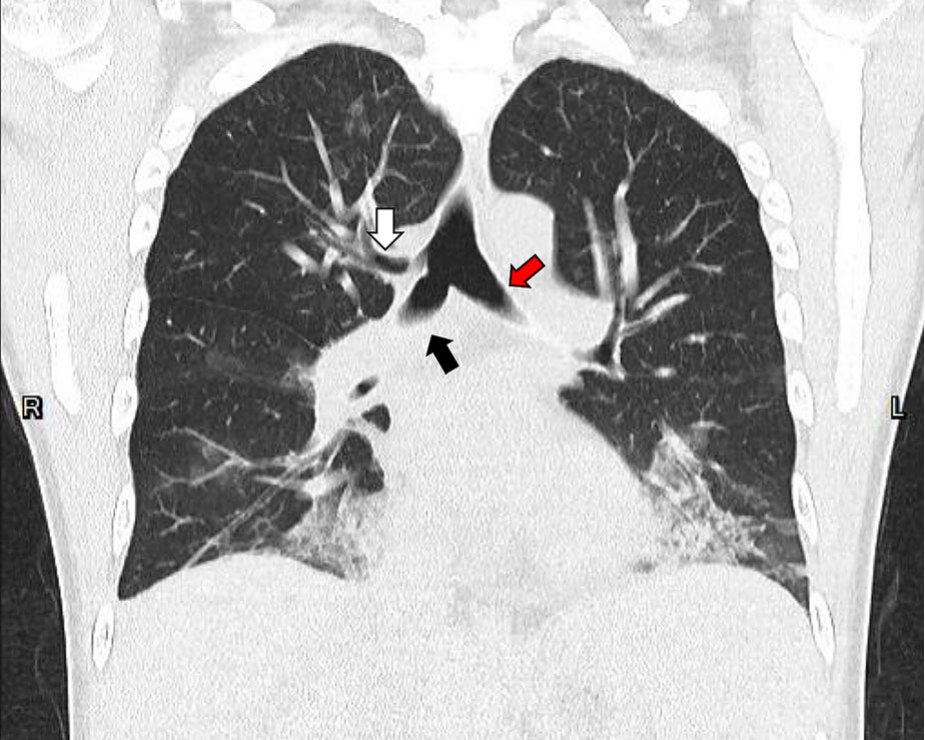
Coronal view of the chest computed tomography with the tracheal bronchus (white arrow), right main bronchus (black arrow) and left main bronchus (red arrow).

**FIGURE 3:**
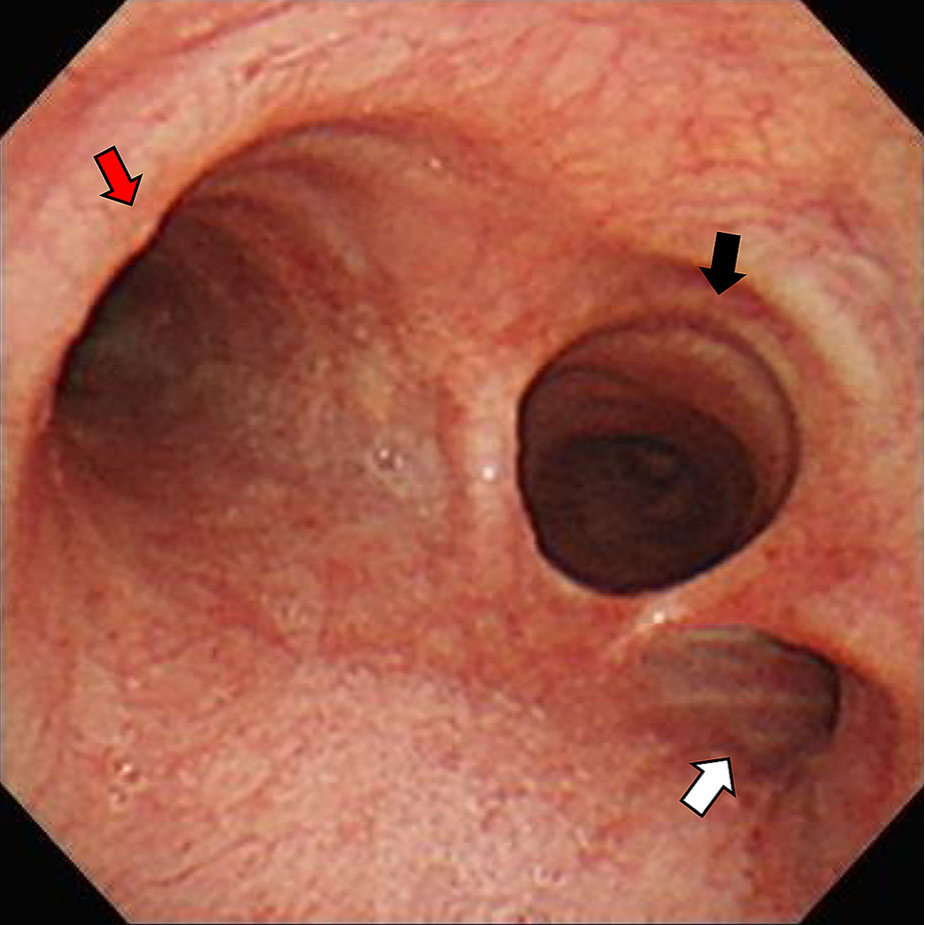
Bronchoscopy revealing orifice of the tracheal bronchus (white arrow) beside the right main bronchus (black arrow) and the orifice of the left main bronchus (red arrow).

A 42‐year‐old man with no systemic disease presented to the emergency department owing to fever and productive cough. Chest radiograph showed bilateral lower lung field consolidation with air bronchogram. Chest computed tomography revealed a suspicion of tracheal bronchus over the distal tracheal region, with absence of the right upper lobe bronchus (Figures 1 and 2). Bronchoscopy was performed, confirming the diagnosis of tracheal bronchus located over the right distal trachea, just above the carina (Figure 3). The site of origin in the right upper lobe bronchus was not found. The patient underwent bronchoalveolar lavage for pathogen identification. His symptoms gradually subsided after appropriate antibiotic treatment.

Congenital anomalies of the large airways are occasionally asymptomatic and are incidentally detected using radiography. The origin sites of the tracheal bronchus usually extend from the lateral wall of the trachea and into the upper lobe. The right sided abnormalities are more common than the left sided ones. The original upper lobe bronchus could be displaced or replaced. Although most patients are asymptomatic, clinical conditions such as bronchial stenosis, stridor, cough, recurrent episodes of pneumonia, or malposition of the endotracheal tube may occur.^1^, ^2^ Physicians should be aware of this anomaly and the possible complications.

## AUTHOR CONTRIBUTIONS

Chia‐Wei Chang wrote the manuscript and processed the image. Chun‐Han Wu performed the manuscript review.

## CONFLICT OF INTEREST STATEMENT

None declared.

## ETHICS STATEMENT

The authors declare that appropriate written informed consent was obtained for the publication of this manuscript and accompanying images.

## Data Availability

The data that support the findings of this study are available on request from the corresponding author. The data are not publicly available due to privacy or ethical restrictions.
